# Supplementary material for: Protective effect of a first SARS-CoV-2 infection from reinfection: a matched retrospective cohort study using PCR testing data in England
Source: Epidemiol Infect. 2022 May 24;150:e109. doi: 10.1017/S0950268822000966 (PMC9171058; doi:10.1017/S0950268822000966)
Supplement: Supplementary file 1 [file S0950268822000966sup001.zip › S2_supp_table1_Sgene.docx]

**Supplementary table S2**  Individuals that had S gene target failure (SGTF) at their second test within population that had a positive PCR test from 15 November onwards (N=3128 with S gene target information, 5307 missing S gene target information) and results of univariable and multivariable logistical regression

|  | Number positive for S gene at second test | Number with S gene target failure (% of total with S gene target) | Number tests after 15 Nov 2020 missing S gene target information (%) | Univariable odds ratio logistic regression for S gene negative | Multivariable odds ratio logistic regression for S gene negative at second test * adjusted for all variables reported |
| --- | --- | --- | --- | --- | --- |
| **First test** |  |  |  |  |  |
| Negative (Control) | 1,632 | 1205 (42.5) | 4358 (60.6) | 1 | 1 |
| Positive (Case) | 132 | 159 (54.6) | 949 (76.5) | 1.63 (1.28 2.08) | 1.9 (1.43 2.51) |
|  |  |  |  |  |  |
| **Region** |  |  |  |  |  |
| London, South East and East of England | 247 | 689 (73.6) | 2541 (73.1) | 1 | 1 |
| Rest of England | 1,517 | 675 (30.8) | 2766 (55.8) | 0.16 (0.13 0.19) | 0.14 (0.11 0.17) |
|  |  |  |  |  |  |
| **Age** |  |  |  |  |  |
| Aged 10-49 years | 1,256 | 1027 (45) | 2823 (55.3) | 1 | 1 |
| Aged 50 and above | 508 | 337 (39.9) | 2484 (74.6) | 0.81 (0.69 0.95) | 0.9 (0.74 1.08) |
|  |  |  |  |  |  |
| **Month of first test** |  |  |  |  |  |
| March | 34 | 30 (46.9) | 222 (77.6) | 0.64 (0.38 1.06) | 0.39 (0.22 0.71) |
| April | 377 | 307 (44.9) | 1591 (69.9) | 0.59 (0.48 0.72) | 0.4 (0.31 0.51) |
| May | 448 | 241 (35) | 1400 (67) | 0.39 (0.32 0.48) | 0.31 (0.24 0.39) |
| June | 188 | 76 (28.8) | 492 (65.1) | 0.29 (0.22 0.4) | 0.23 (0.16 0.32) |
| July | 149 | 98 (39.7) | 313 (55.9) | 0.48 (0.36 0.64) | 0.41 (0.29 0.57) |
| August | 238 | 156 (39.6) | 415 (51.3) | 0.47 (0.37 0.61) | 0.37 (0.28 0.49) |
| September | 330 | 456 (58) | 874 (52.7) | 1 | 1 |
|  |  |  |  |  |  |
| **Ethnicity** |  |  |  |  |  |
| White | 1,258 | 953 (43.1) | 3926 (64) | 1 | 1 |
| Asian or Asian British | 283 | 195 (40.8) | 515 (51.9) | 0.91 (0.74 1.11) | 0.76 (0.61 0.96) |
| Black or Black British | 38 | 48 (55.8) | 208 (70.7) | 1.67 (1.08 2.57) | 1.05 (0.65 1.72) |
| Mixed | 17 | 24 (58.5) | 81 (66.4) | 1.86 (1 3.49) | 1.21 (0.59 2.48) |
| Other ethnic groups | 33 | 46 (58.2) | 155 (66.2) | 1.84 (1.17 2.9) | 0.9 (0.53 1.51) |
| Missing | 135 | 98 (42.1) | 422 (64.4) |  |  |
|  |  |  |  |  |  |
| **First test route** |  |  |  |  |  |
| Hospital testing | 424 | 350 (45.2) | 2431 (75.9) | 1 |  |
| Community testing | 1,340 | 1014 (43.1) | 2876 (55) | 0.92 (0.78 1.08) |  |
|  |  |  |  |  |  |
| **Sex** |  |  |  |  |  |
| F | 1,143 | 835 (42.2) | 3565 (64.3) | 1 |  |
| M | 621 | 529 (46) | 1742 (60.2) | 1.17 (1.01 1.35) |  |
|  |  |  |  |  |  |
| **IMD quintiles** |  |  |  |  |  |
| 1 | 564 | 347 (38.1) | 1235 (57.5) | 1 |  |
| 2 | 394 | 312 (44.2) | 1197 (62.9) | 1.29 (1.05 1.57) |  |
| 3 | 318 | 284 (47.2) | 1092 (64.5) | 1.45 (1.18 1.79) |  |
| 4 | 260 | 229 (46.8) | 988 (66.9) | 1.43 (1.15 1.79) |  |
| 5 | 226 | 189 (45.5) | 781 (65.3) | 1.36 (1.07 1.72) |  |
| Missing | 2 | 3 (60) | 14 (73.7) |  |  |
