# Supplementary material for: Protective effect of a first SARS-CoV-2 infection from reinfection: a matched retrospective cohort study using PCR testing data in England
Source: Epidemiol Infect. 2022 May 24;150:e109. doi: 10.1017/S0950268822000966 (PMC9171058; doi:10.1017/S0950268822000966)
Supplement: Supplementary file 1 [file S0950268822000966sup001.zip › S1_supp_table1_symptoms.docx]

**Supplementary table S1**  Individuals that had symptoms at their second test within population that had a positive PCR test (post hoc population) during the follow up period and results of univariable and multivariable logistical regression

|  | Number asymptomatic at positive test | Number with symptoms at positive test (% of total with symptom information) | Number with missing symptom information at second test (%) | Univariable odds ratio logistic regression for symptoms at second test | Multivariable odds ratio logistic regression for symptoms at second test * adjusted for all variables reported |
| --- | --- | --- | --- | --- | --- |
| **First test** |  |  |  |  |  |
| Negative (Control) | 2,983 | 5860 (66.3) | 2875 (24.5) | 1 | 1 |
| Positive (Case) | 1,163 | 499 (30) | 599 (26.5) | 0.22 (0.19 0.24) | 0.23 (0.2 0.26) |
|  |  |  |  |  |  |
| **Sex** |  |  |  |  |  |
| F | 3,075 | 3948 (56.2) | 2256 (24.3) | 1 | 1 |
| M | 1,071 | 2411 (69.2) | 1218 (25.9) | 1.75 (1.61 1.91) | 1.67 (1.51 1.84) |
|  |  |  |  |  |  |
| **Age** |  |  |  |  |  |
| Aged 10-49 years | 2,005 | 4728 (70.2) | 1482 (18) | 1 | 1 |
| Aged 50 and above | 2,141 | 1631 (43.2) | 1992 (34.6) | 0.32 (0.3 0.35) | 0.33 (0.3 0.36) |
|  |  |  |  |  |  |
| **Month of first test** |  |  |  |  |  |
| March | 65 | 146 (69.2) | 260 (55.2) | 1.17 (0.86 1.6) | 2.14 (1.48 3.08) |
| April | 970 | 1687 (63.5) | 1566 (37.1) | 0.91 (0.79 1.04) | 1.32 (1.14 1.54) |
| May | 1,535 | 1715 (52.8) | 944 (22.5) | 0.58 (0.51 0.66) | 0.84 (0.73 0.97) |
| June | 542 | 747 (58) | 312 (19.5) | 0.72 (0.62 0.84) | 0.86 (0.72 1.02) |
| July | 254 | 536 (67.8) | 150 (16) | 1.1 (0.92 1.32) | 1.19 (0.97 1.47) |
| August | 260 | 531 (67.1) | 99 (11.1) | 1.07 (0.89 1.28) | 1.2 (0.98 1.48) |
| September | 520 | 997 (65.7) | 143 (8.6) | 1 | 1 |
|  |  |  |  |  |  |
| **Region** |  |  |  |  |  |
| London | 313 | 803 (72) | 427 (27.7) | 1 | 1 |
| East Midlands | 529 | 681 (56.3) | 275 (18.5) | 0.5 (0.42 0.6) | 0.56 (0.46 0.69) |
| East of England | 369 | 490 (57) | 351 (29) | 0.52 (0.43 0.62) | 0.62 (0.5 0.77) |
| North East | 253 | 372 (59.5) | 360 (36.5) | 0.57 (0.47 0.7) | 0.69 (0.54 0.87) |
| North West | 805 | 1509 (65.2) | 754 (24.6) | 0.73 (0.63 0.85) | 0.79 (0.65 0.94) |
| South East | 583 | 640 (52.3) | 496 (28.9) | 0.43 (0.36 0.51) | 0.47 (0.38 0.57) |
| South West | 204 | 190 (48.2) | 142 (26.5) | 0.36 (0.29 0.46) | 0.43 (0.33 0.57) |
| West Midlands | 514 | 673 (56.7) | 376 (24.1) | 0.51 (0.43 0.61) | 0.56 (0.46 0.69) |
| Yorkshire and Humber | 576 | 1001 (63.5) | 293 (15.7) | 0.68 (0.57 0.8) | 0.72 (0.59 0.87) |
|  |  |  |  |  |  |
| **Ethnicity** |  |  |  |  |  |
| White | 3,210 | 4548 (58.6) | 2560 (24.8) | 1 | 1 |
| Asian or Asian British | 305 | 917 (75) | 332 (21.4) | 2.12 (1.85 2.43) | 1.56 (1.34 1.81) |
| Black or Black British | 173 | 152 (46.8) | 110 (25.3) | 0.62 (0.5 0.77) | 0.44 (0.34 0.56) |
| Mixed | 57 | 103 (64.4) | 40 (20) | 1.28 (0.92 1.77) | 0.97 (0.68 1.39) |
| Other ethnic groups | 81 | 169 (67.6) | 91 (26.7) | 1.47 (1.13 1.93) | 0.97 (0.72 1.31) |
| Missing | 320 | 470 (59.5) | 341 (30.2) |  |  |
|  |  |  |  |  |  |
| **IMD quintiles** |  |  |  |  |  |
| 1 | 1,171 | 1780 (60.3) | 915 (23.7) | 1 |  |
| 2 | 950 | 1419 (59.9) | 789 (25) | 0.98 (0.88 1.1) |  |
| 3 | 782 | 1161 (59.8) | 660 (25.4) | 0.98 (0.87 1.1) |  |
| 4 | 693 | 1087 (61.1) | 623 (25.9) | 1.03 (0.91 1.16) |  |
| 5 | 539 | 901 (62.6) | 481 (25) | 1.1 (0.97 1.25) |  |
| Missing | 11 | 11 (50) | 6 (21.4) |  |  |
|  |  |  |  |  |  |
| **First test route** |  |  |  |  |  |
| Hospital testing | 1,111 | 1760 (61.3) | 2686 (48.3) | 1 |  |
| Community testing | 3,035 | 4599 (60.2) | 788 (9.4) | 0.96 (0.88 1.04) |  |
|  |  |  |  |  |  |
