# Supplementary figures and images for: Protective effect of a first SARS-CoV-2 infection from reinfection: a matched retrospective cohort study using PCR testing data in England
Source: Epidemiol Infect. 2022 May 24;150:e109. doi: 10.1017/S0950268822000966 (PMC9171058; doi:10.1017/S0950268822000966)

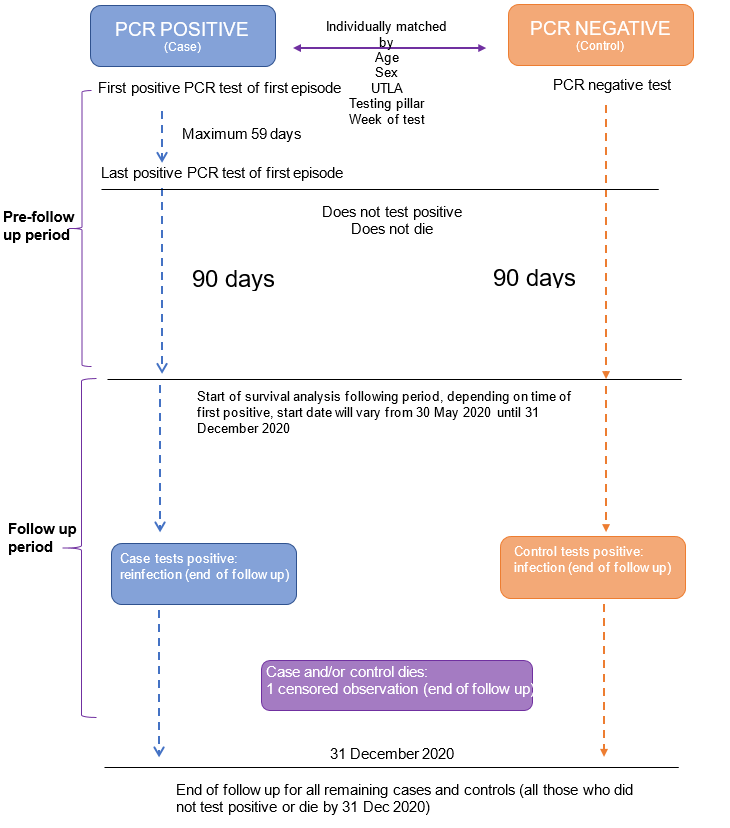

Supplement: Supplementary file 1 [file S0950268822000966sup001.zip › Supplmentary_fig_1.PNG]
